# Preoperative Right Ventricular Tei Index as a High-Risk Marker for Adverse Outcomes in Patients Undergoing Cardiac Surgery

**DOI:** 10.5761/atcs.oa.25-00149

**Published:** 2025-12-02

**Authors:** Araceli González-Ortiz, Daniel Manzur-Sandoval, José Antonio Arias-Godínez, Edith Liliana Posada-Martínez, Edgar García-Cruz, Rodrigo Gopar-Nieto, Gustavo Rojas-Velasco

**Affiliations:** 1 Cardiology Department, Ignacio Chávez National Institute of Cardiology, Mexico City, Mexico; 2 Cardiovascular Critical Care Unit, Ignacio Chávez National Institute of Cardiology, Mexico City, Mexico; 3 Echocardiography Laboratory, Ignacio Chávez National Institute of Cardiology, Mexico City, Mexico; 4 Adult Congenital Heart Disease Unit, Ignacio Chávez National Institute of Cardiology, Mexico City, Mexico; 5 Coronary Care Unit, Ignacio Chávez National Institute of Cardiology, Mexico City, Mexico

**Keywords:** right ventricular function, Tei index, hemodynamic monitoring, cardiac surgery

## Abstract

**Purpose:**

Cardiovascular surgery entails considerable risk because of its complexity and the frequency of perioperative complications. The right ventricular Tei index (RV-TI) provides an integrated measure of ventricular performance, encompassing systolic and diastolic function. Although not widely applied in this setting, its role as a predictor of outcomes is promising. This study evaluated the utility of RV-TI in predicting postoperative complications in patients undergoing diverse cardiovascular surgeries, emphasizing its value for surgical risk stratification and patient management.

**Methods:**

A single-center, cross-sectional study was conducted at the National Institute of Cardiology Ignacio Chávez, Mexico City, including 195 adults who underwent cardiac surgery between June 2022 and April 2023. RV-TI was obtained by transthoracic tissue Doppler, using 0.53 as the abnormal cutoff. One hundred and forty-nine patients had normal RV-TI values, while 46 were classified as abnormal.

**Results:**

Abnormal RV-TI correlated with diabetes mellitus, advanced age, and vasoplegic syndrome. These patients experienced higher in-hospital mortality and more severe complications, including the need for renal replacement therapy, pneumonia, delirium, and greater transfusion requirements.

**Conclusion:**

RV-TI appears to be a valuable adjunct in preoperative risk assessment for cardiac surgery. Its incorporation into clinical practice could improve patient selection and decision-making, contributing to better surgical outcomes.

## Introduction

### Background

Cardiovascular diseases represent a significant global health burden, necessitating complex surgical interventions for conditions such as coronary artery disease, valvular pathologies, and advanced heart failure. These surgeries entail considerable risk of postoperative complications and adverse outcomes, prompting rigorous preoperative risk assessment to identify high-risk patients and optimize management strategies.^1,2)^

### Importance

Effective risk assessment in cardiovascular surgery is critical to mitigate complications and mortality. Current risk models like the STS (Society of Thoracic Surgeons) score and EuroSCORE II incorporate limited echocardiographic parameters focused mainly on left ventricular systolic function. However, echocardiography offers broader insights into ventricular function, potentially enhancing risk prediction accuracy.^3)^ The right ventricular Tei index (RV-TI), a measure combining systolic and diastolic parameters, presents an opportunity to refine risk assessment tools in this patient population.

The TI is a noninvasive echocardiographic measure designed to evaluate overall ventricular function by integrating both systolic and diastolic parameters. It is calculated as the ratio of the sum of isovolumetric relaxation time (IVRT) and isovolumetric contraction time (IVCT) to the ejection time (ET):

• TI = (IVRT + IVCT)/ET

**Fig. 1:**
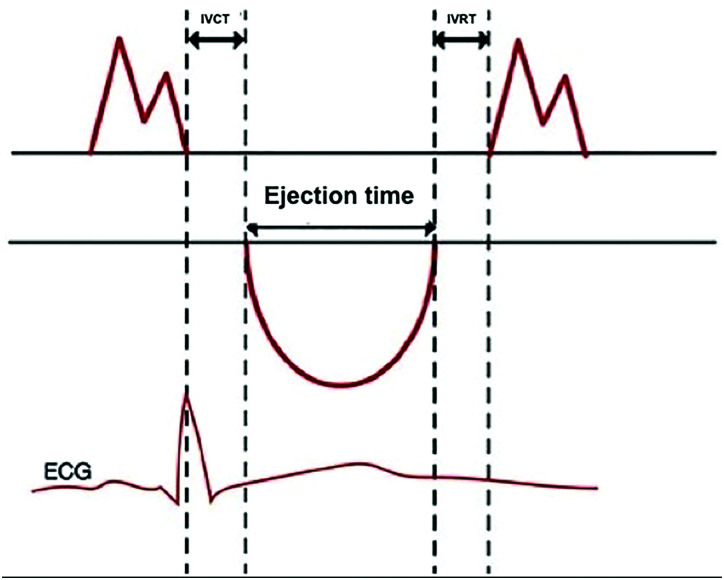
Tei index. IVRT: isovolumetric relaxation time; IVCT: isovolumetric contraction time; ECG: electrocardiogram. Adapted from reference 4. © 2016 Grupo CTO. Licensed under CC BY-NC-ND 4.0.

This index is illustrated in relation to the electrocardiogram in **Fig. 1**. In cases of systolic ventricular dysfunction, there is a shortened ET relative to the isovolumetric phases. Conversely, diastolic dysfunction manifests as impaired ventricular filling, accompanied by lengthened relaxation periods. The normal range of the TI may vary slightly with age, but typically a value of 0.40 or lower is considered normal in adults.^4)^

Limited research currently assesses the effectiveness of the TI in predicting cardiovascular outcomes among cardiac surgery patients. The majority of the information currently available in the medical literature relates to left ventricular TI (LV-TI). Nevertheless, existing data suggest that a high TI can effectively predict an increased risk of postsurgical complications, prolonged hospitalization, and other adverse events.

For instance, studies conducted in various countries provide insights into the LV-TI’s predictive capabilities. In France, a study involving 25 patients diagnosed with mitral insufficiency prior to valve replacement found that the TI was more accurate than fractional area change (FAC) in identifying patients with ventricular dysfunction who might struggle to wean off cardiopulmonary bypass.^5)^ Similarly, research in Japan involving 154 patients with severe aortic stenosis demonstrated higher postsurgical mortality among those with abnormal LV-TI before valve replacement.^6)^ Conversely, a study in Germany with 104 patients undergoing valve replacement for severe mitral insufficiency did not find the preoperative LV-TI to be a reliable predictor of changes in postsurgical cardiac function metrics.^7)^ A 2019 study by the American Heart Association involving 824 patients eligible for transcatheter aortic valve replacement revealed that a high preoperative LV-TI correlated with increased mortality at 30 days and one year post-procedure.^8)^

There is very limited information in the literature regarding the clinical use and predictive capacity of RV-TI. A study in Spain involving 26 patients undergoing various cardiac surgeries compared the TI with parameters such as systolic excursion of the tricuspid annular plane systolic excursion (TAPSE) and tricuspid annular peak systolic velocity (S wave). It demonstrated that the TI exhibited minimal postoperative changes without statistically significant correlations with extracorporeal circulation or complication risk.^9)^

Based on current evidence, TI is a noninvasive echocardiographic measure that may help identify patients at higher risk of mortality and complications in cardiac surgery and interventional cardiology. While most of the available literature has focused on assessing global LV function using LV-TI, there is very limited evidence regarding the clinical utility and predictive capacity of RV-TI. This scarcity of data strengthens the relevance of our study, as it addresses an underexplored parameter with potential prognostic value.

### Investigation goals

The study aimed to establish the efficacy of RV-TI in predicting perioperative outcomes, potentially enhancing surgical risk stratification and clinical decision-making in cardiovascular surgery.

## Materials and Methods

### Study design

This was a single-center, observational, cross-sectional, retrospective study.

### Study population

The study included adult patients admitted to the Postoperative Intensive Care Unit of the National Institute of Cardiology Ignacio Chávez who underwent cardiac surgery between June 1, 2022 and April 30, 2023.

### Inclusion criteria

The inclusion criteria included patients aged 18 years and older who underwent cardiac surgery with cardiopulmonary bypass and had a stay of at least 12 hours in the postoperative intensive care unit.

### Exclusion criteria

In the exclusion criteria, patients were excluded if they did not undergo a preoperative transthoracic echocardiogram for any reason, if tricuspid tissue Doppler was not obtained during echocardiography, or if they had factors that made tricuspid inflow measurement unreliable (such as atrial fibrillation or flutter, tricuspid valve prosthesis, or complex congenital heart diseases like single ventricle patients). Additionally, patients with fatal outcomes in the operating room were excluded.

All procedures included in this study were performed under cardiopulmonary bypass with central cannulation and aortic cross-clamping. Myocardial protection was achieved primarily with antegrade cardioplegia using Del Nido solution administered after aortic cross-clamping through the aortic root, prepared in a 1:4 ratio of crystalloid to blood (20%/80%) and cooled to 4°C–10°C. The initial dose was 20 mL/kg (approximately 1000 mL), which provided 60–90 min of myocardial protection; for longer ischemic times, supplemental doses of 10 mL/kg were given every 60–90 min. In selected cases, such as proximal coronary obstructions or complex valvular procedures, retrograde administration through the coronary sinus was added as an adjunct, but never used as the sole delivery method. This strategy ensured effective metabolic and electrical protection, with reduced total volume and minimized manipulation of the aortic root. Systemic hypothermia was maintained according to the current institutional protocol, targeting a core temperature of 32°C–34°C during cardiopulmonary bypass. This approach ensured optimal myocardial protection, particularly in the right coronary distribution, and minimized potential confounding effects associated with retrograde perfusion.

### Ultrasound equipment

The operator obtained the images using a phased-array sector probe at 2–3 mHz, from the patient’s right or left side, on any platform with the following modes: M-mode, 2D-mode, color, pulsed wave (PW), continuous wave (CW), and tissue Doppler imaging (TDI).

The following parameters were measured:

LV ejection fraction (LVEF).

Diastolic dysfunction:

• Grade 1—Impaired relaxation:
  ○ Mitral inflow: E/A ratio <0.8
  ○ Deceleration time (DT) >200 ms
  ○ Isovolumic relaxation time (IVRT) prolonged
  ○ Left atrial (LA) volume is normal or mildly increased
  ○ Pulmonary vein systolic/diastolic (S/D) ratio is normal or slightly reversed
  ○ TDI: e′ velocity is reduced (<age-specific reference), and an E/e′ ratio usually <10
• Grade 2—Pseudonormal filling:
  ○ Mitral inflow: E/A ratio 0.8–1.5 (may appear normal)
  ○ DT 160–200 ms
  ○ LA volume is moderately increased
  ○ Pulmonary vein S/D ratio shows blunted systolic filling
  ○ TDI e′ velocity reduced and an E/e′ ratio 10–14
  ○ Valsalva maneuver unmasking: E/A ratio decreases <1
• Grade 3—Restrictive filling:
  ○ Mitral inflow: E/A ratio ≥2
  ○ DT <160 ms
  ○ LA volume markedly increased
  ○ Pulmonary vein: Systolic flow decreased and diastolic flow being dominant
  ○ TDI: Markedly reduced e′ and E/e′ ratio >14
  ○ Often reversible with preload reduction (reversible restrictive) or fixed (irreversible)

### RV function: FAC, TAPSE, and S wave

The echocardiographic views and parameters were recorded and measured according to the guidelines of the American Society of Echocardiography for performing a comprehensive transthoracic echocardiographic examination in adults, as well as the guidelines of the American Society of Echocardiography and the European Association of Cardiovascular Imaging for cardiac chamber quantification.

### RV-TI measurement

The TI can be obtained using either PW Doppler or TDI. The main limitation of PW Doppler is that measurements cannot be obtained within the same cardiac cycle. For the RV-TI, inflow at the tricuspid valve is measured in the apical 4-chamber view, while outflow from the RV outflow tract requires a separate projection, such as the 5-chamber view. In contrast, TDI allows these measurements to be acquired from a single image acquisition, improving consistency and reproducibility; therefore we chose to use the latter. To calculate the TI using TDI:[Fig F2]

**Fig. 2 F2:**
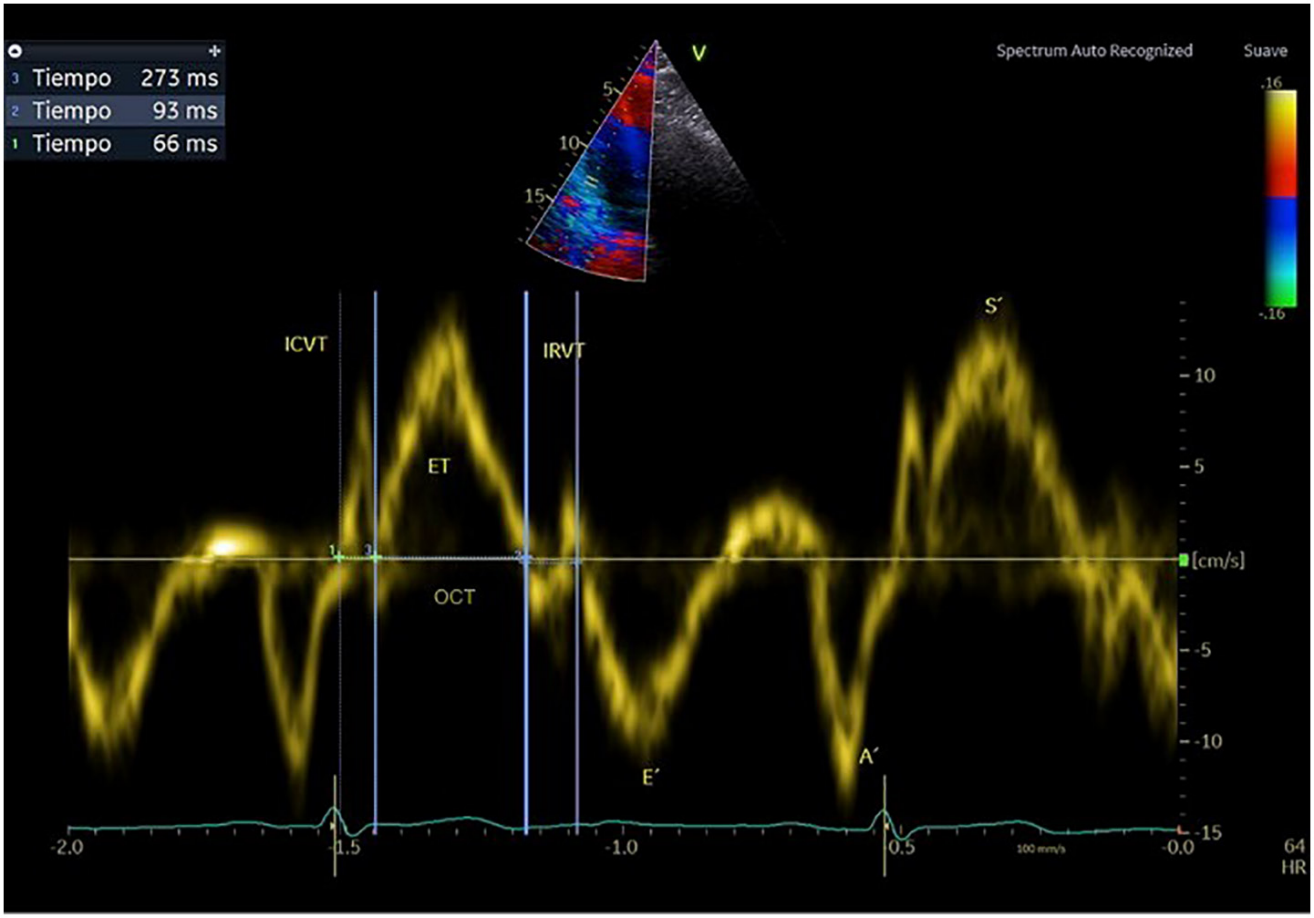
Tricuspid tissue Doppler velocity recording in apical 4-chamber view. E′: early diastolic wave; A′: late diastolic wave; S′: systolic wave. IVCT: isovolumetric contraction time; ET: ejection time; IVRT: isovolumetric relaxation time; OCT: opening and closing time

• The sample transducer from the apical 4-chamber view is placed at the lateral end of the tricuspid annulus.
• The time interval is measured from the end of the late diastolic wave velocity A′ to the beginning of the E′ wave, representing the time from tricuspid valve closure to opening (tricuspid annulus contraction time, TAC). This period includes IVCT, ET, and IVRT, and is visually represented in **Fig. 2**.^4)^ Schematically, we can represent the calculation of the RV-TI as follows:
  • TI = (TAC − ET)/ET = (IVCT + IVRT)/ET

Images were processed and analyzed after acquisition. One physician (a critical care physician with training in critical care ultrasonography) reviewed the images, after which the images were processed and measured by 3 different physicians (clinical cardiologists with training in echocardiography: AG-O, JAA-G, and ELP-M) using imaging software (Siemens Healthineers, Erlangen, Germany). We reduced bias by blinding the investigators who analyzed, processed, and measured the images.

Current guidelines for the quantification of RV function define normal values of the RV-TI index as <0.40 when measured by PW Doppler and <0.55 when measured by TDI.^10)^ In our study, however, we deliberately chose not to directly adopt these cut-off values. Given that our cohort consisted exclusively of patients with surgical cardiovascular pathology, we considered it more appropriate to establish a population-specific threshold. Because the distribution of our data was non-normal, we used the median RV-TI index and defined the upper quartile (75th percentile) as the cut-off, which in our population corresponded to 0.53. This value is very close to the guideline-reported cut-off for the TDI method, supporting its suitability and validity for our analysis.

In all patients, central venous pressure (CVP) was measured invasively using a central venous catheter with the tip at the cavoatrial junction. All patients were also monitored invasively with a pulmonary artery catheter. We performed serial measurements in the first hours (on admission and at 6 and 24 hours) of the postoperative period after cardiac surgery. We measured macrocirculatory parameters, global oxygenation indices, CO_2_-derived indices, and lactate levels.

### Definition of postoperative clinical syndromes^11)^

Low cardiac output syndrome:

• Cardiac index <2.2 L/min/m^2^, with systolic blood pressure <90 mmHg and pulmonary artery occlusion pressure (PAOP) ≥16 mmHg and/or CVP ≥12 mmHg.
• Urine output <0.5 mL/kg/h, central venous oxygen saturation <60%, and lactate >3 mmol/L.
• Postoperative use of inotropes and/or ventricular assist devices for at least 12 hours.
• Real or relative hypovolemia was ruled out or managed, with no predictors of fluid responsiveness.

Vasoplegic syndrome:

• Hypotension:
  ○ Systolic blood pressure <80 mmHg.
  ○ Mean arterial pressure <50 mmHg.
• Vasodilation:
  ○ Systemic vascular resistance <800 dynes/s/cm^5^.
• CVP >5 mmHg.
• PAOP >10 mmHg.
• Cardiac index >2.2 L/min/m^2^.
• Use of vasopressors:
  ○ Norepinephrine >0.3 μg/kg/min.
• Real or relative hypovolemia was ruled out or managed, with no predictors of fluid responsiveness.

Excessive bleeding:

➢ 300 mL in the first hour after surgery.
➢ 200 mL/hour for more than 2 hours.
➢ 100 mL/hour for more than 3 hours.

**Fig. 3:**
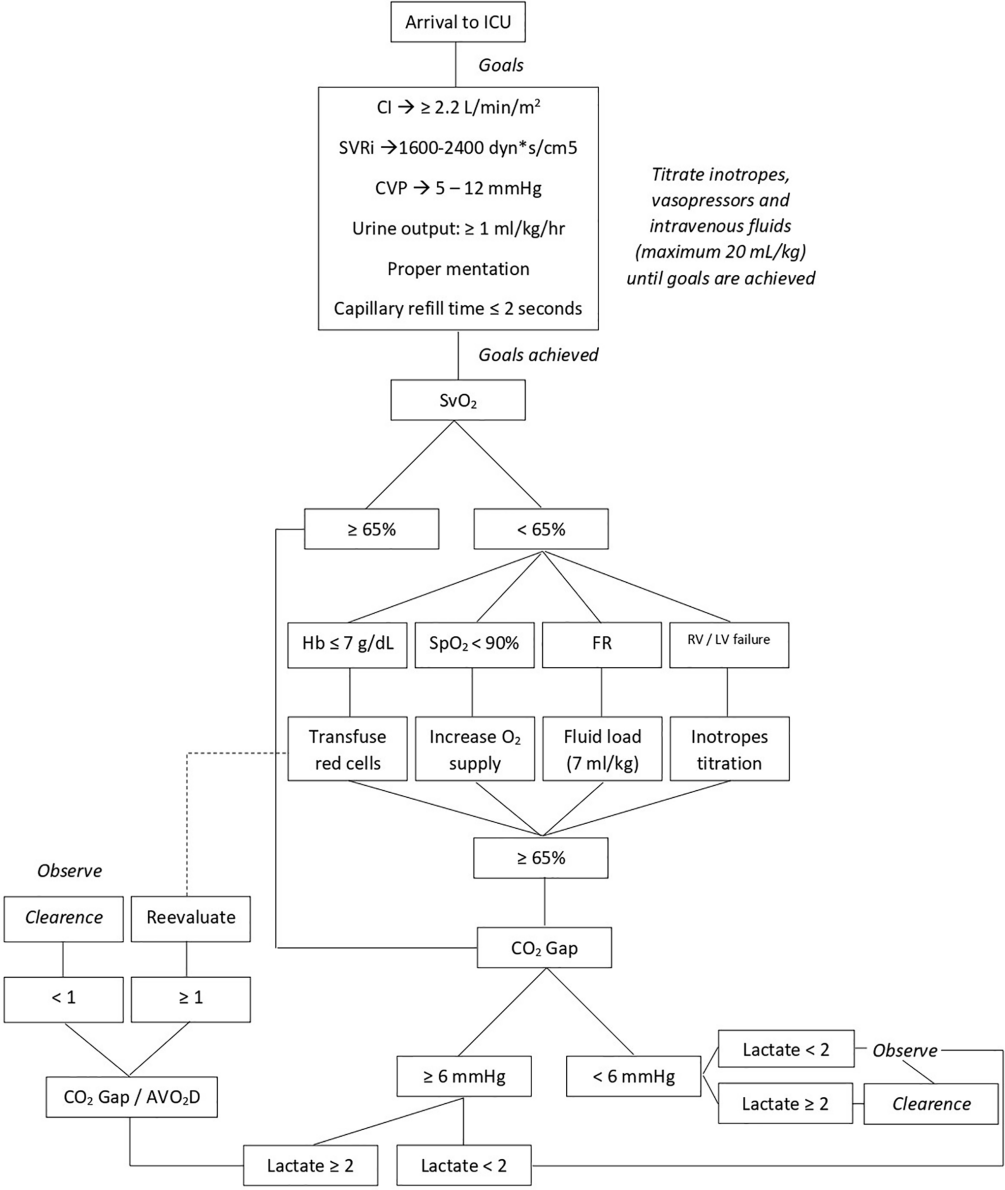
Hemodynamic assessment and vasoactive drug titration. Monitoring of CI, SVRi, CVP, SvO_2_, Hb, SpO_2_, FR, RV and LV function, CO_2_ gap, and AVO_2_D guided therapy. CI: cardiac index; SVRi: systemic vascular resistance index; CVP: central venous pressure; SvO_2_: mixed venous oxygen saturation; Hb: hemoglobin; SpO_2_: peripheral oxygen saturation; FR: fluid responsiveness; RV: right ventricle; LV: left ventricle; CO_2_ gap: central venous-to-arterial carbon dioxide difference; AVO_2_D: arteriovenous oxygen difference

The methods for hemodynamic assessment and optimization, as well as for vasoactive drug titration, are illustrated in **Fig. 3**.^12)^

### Statistical analysis

The Shapiro–Wilk test of normality was used for continuous variables. Parametric variables were reported as mean ± standard deviation, and non-parametric variables were reported as median with interquartile range (IQR). Continuous variables were compared using the Mann–Whitney U test. Categorical variables were described using frequencies and percentages. The chi-squared and Fisher’s exact tests were used for comparisons based on expected values. All postoperative clinical outcomes were initially compared between patients with normal and abnormal RV-TI (≥0.53) to identify statistically significant differences between the groups. Outcomes analyzed included delirium, stroke, in-hospital pneumonia, mediastinitis, transfusion, acute kidney injury (AKI), renal replacement therapy, liver injury, postoperative atrial fibrillation, and in-hospital mortality. Only outcomes showing statistically significant differences between the groups were included in univariate logistic regression analyses. Variables demonstrating clinical relevance or statistical significance in univariate analyses (p <0.05) were then included in multivariate logistic regression models to determine independent predictors. Age and diabetes were included as adjustment variables because both showed statistically significant differences between groups. Sex was also included given its established influence on ventricular function and postoperative prognosis. Diabetes was considered particularly relevant as a cardiovascular risk factor in the context of diastolic dysfunction. Results are presented as odds ratios (ORs) with 95% confidence intervals (CIs), and statistical significance was defined as p <0.05. All analyses were performed using STATA version 14 (StataCorp, College Station, TX, USA).

## Results

### Baseline characteristics

A total of 195 adults underwent surgical intervention and were studied. The gender distribution was 43.6% females and 56.4% males. The median age was 59 years, with patients in the abnormal RV-TI group being older (median 62 years, p = 0.03). The median body mass index was 25.9 kg/m^2^. Prior to surgery, most patients were in New York Heart Association (NYHA) functional class II (49.2%), followed by NYHA class III (30.8%), NYHA class I (17.4%), and NYHA class IV (2.6%). Approximately 18% had a history of prior cardiac surgery.

The prevalence of chronic-degenerative diseases was as follows: hypothyroidism (22%), previous myocardial infarction (17%), diabetes mellitus (49%), chronic kidney disease (4%), systemic arterial hypertension (84%), previous stroke (9%), and heart failure (39%). Diabetes mellitus was significantly more prevalent among patients with abnormal RV-TI (p = 0.03).

The EuroSCORE II-calculated surgical risk showed no significant differences between groups (median 1.6 points). Additional echocardiographic variables evaluating systolic function were similar between the normal and abnormal RV-TI groups: median LVEF 56%, TAPSE 21 mm, FAC 43%, and S wave 11 cm/s.

Regarding LV diastolic function, 52.1% of patients had grade I dysfunction, 22.5% had grade II dysfunction, and 25.3% had grade III dysfunction (**Table 1**).

**Table 1:** Baseline characteristics

Variable	Total (n = 195)	RV-TI <0.53 (n = 149)	RV-TI ≥0.53 (n = 46)	p value
Women, N (%)	85 (43.6)	66 (44.3)	19 (41.3)	0.72
Men, N (%)	110 (56.4)	83 (55.7)	27 (58.7)	
Age (years), median (IQR)	59 (48–66)	57 (45–65)	62 (55–67)	0.03
Height (m), median (IQR)	1.62 (1.54–1.7)	1.62 (1.54–1.7)	1.62 (1.56–1.68)	0.78
Weight (kg), median (IQR)	69.7 (59–78)	70 (59–78)	68.2 (59.6–73)	0.30
Body mass index (kg/m^2^), median (IQR)	25.9 (23.3–28.4)	25.9 (23.4–28.4)	25.6 (22.8–27.6)	0.33
NYHA functional class, N (%)				
I	34 (17.4)	27 (18.1)	7 (15.2)	0.59
II	96 (49.2)	74 (49.7)	22 (47.8)	
III	60 (30.8)	43 (28.9)	17 (37)	
IV	5 (2.6)	5 (3.4)	0	
Previous cardiac surgery, N (%)	18 (9.2)	13 (8.7)	5 (10.9)	0.77
Hypothyroidism, N (%)	22 (11.3)	19 (12.7)	3 (6.5)	0.29
Myocardial infarction, N (%)	17 (8.7)	12 (8)	5 (10.9)	0.55
Diabetes, N (%)	49 (25.1)	32 (21.5)	17 (37)	0.03
Chronic kidney disease, N (%)	4 (2)	2 (1.3)	2 (4.3)	0.23
Hypertension, N (%)	84 (43.1)	64 (42.9)	20 (43.5)	0.95
Cerebrovascular disease, N (%)	9 (4.6)	7 (4.7)	2 (4.3)	1
Heart failure, N (%)	39 (20)	32 (21.5)	7 (15.2)	0.35
EuroSCORE II, median (IQR)	1.6 (0.8–3.2)	1.6 (0.8–3.2)	1.4 (0.8–3.2)	0.82
LVEF (%), median (IQR)	56 (48–60)	56 (48–61)	56 (47–60)	0.96
Diastolic dysfunction (grade), N (%)				
I	74 (52.1)	54 (51.9)	20 (52.6)	0.72
II	32 (22.5)	25 (24)	7 (18.4)	
III	36 (25.3)	25 (24)	11 (28.9)	
TAPSE (mm), median (IQR)	21 (18–23)	21 (18–24)	21 (18–22)	0.71
FACVD (%), median (IQR)	43 (36–50)	43 (36–50)	43 (38–49)	0.69
S wave, median (IQR)	11 (9.6–12.5)	11 (9.6–12.5)	11.2 (9.7–12.5)	0.73

RV-TI: Right ventricular Tei index; NYHA: New York Heart Association; IQR: interquartile range; LVEF: left ventricular ejection fraction; TAPSE: tricuspid annular plane systolic excursion; FACVD: fractional area change of the right ventricular outflow tract

### Surgical characteristics

Among the types of cardiac surgery performed, the distribution was as follows: aortic valve replacement accounted for 27.2%, coronary artery bypass grafting for 16.4%, mitral valve replacement for 10.3%, mitral valve combined with tricuspid valve replacement for 5.1%, aortic valve combined with mitral valve replacement for 6.7%, combined revascularization with aortic valve replacement for 5.1%, Bentall and Bono surgery for 5.6%, and other types of surgery for 23.6%. There were no significant differences in surgical times between both groups, with a median cardiopulmonary bypass time of 144 min and an aortic cross-clamp time of 99 min. Various immediate postoperative complications were reported: major mediastinal bleeding occurred in 13.3% of cases, postcardiotomy low cardiac output syndrome in 9.2%, vasoplegic syndrome in 7.2%, and hypovolemia in 36.9%. Significant differences were observed in vasoplegic syndrome between groups, with a higher frequency in the abnormal RV-TI group (15.2% versus 4.7%, p = 0.01) (**Table 2**).

**Table 2:** Surgical characteristics

Variable	Total (n = 195)	RV-TI <0.53 (n = 149)	RV-TI ≥0.53 (n = 46)	p value
Aortic valve replacement, N (%)	53 (27.2)	42 (28.2)	11 (23.9)	0.56
Coronary artery bypass graft, N (%)	32 (16.4)	21 (14.1)	11 (23.9)	0.11
Mitral valve replacement, N (%)	20 (10.3)	14 (9.4)	6 (13)	0.47
Mitral valve replacement + tricuspid valve replacement, N (%)	10 (5.1)	8 (5.4)	2 (4.3)	1
Aortic valve replacement + mitral valve replacement, N (%)	13 (6.7)	11 (7.4)	2 (4.3)	0.73
Coronary artery bypass graft + aortic valve replacement, N (%)	10 (5.1)	8 (5.4)	2 (4.3)	1
Bentall procedure, N (%)	11 (5.6)	9 (6)	2 (4.3)	1
Other surgery, N (%)	46 (23.6)	36 (24.2)	10 (21.7)	0.73
Mediastinal bleeding, N (%)	26 (13.3)	21 (14.1)	5 (10.9)	0.80
Low cardiac output syndrome, N (%)	18 (9.2)	14 (9.4)	4 (8.7)	1
Vasoplegic syndrome, N (%)	14 (7.2)	7 (4.7)	7 (15.2)	0.01
Hypovolemia, N (%)	72 (36.9)	58 (38.9)	14 (30.4)	0.29
Extracorporeal circulation time (min), median (IQR)	144 (113–192)	143 (114–189)	151 (109–198)	0.61
Aortic clamping (min), median (IQR)	99 (78–128)	101 (81–127)	91 (73–129)	0.53

RV-TI: right ventricular Tei index; IQR: interquartile range

### Hemodynamic parameters and vasoactive drugs

The hemodynamic evolution of patients was analyzed at 6 and 24 hours during their stay in the postoperative intensive care unit. Significant differences were noted in the mixed venous O_2_ saturation at 24 hours, which was lower in the group with abnormal RV-TI, with a median of 64% versus 68% in the normal RV-TI group (p <0.001). Additionally, a higher O_2_ extraction ratio at 24 hours was found in the abnormal RV-TI group, with a median of 33% versus 30% in the normal RV-TI group (p <0.001). Serum lactate levels at 24 hours were significantly higher in patients with abnormal RV-TI, with a median of 2.5 versus 2.0 mmol/L (p = 0.01).

At 6 hours, a significant difference was found in the requirement for norepinephrine, which was more frequent among patients with abnormal RV-TI, required in 71.7% of cases compared with 51.7% in those with normal RV-TI (p = 0.01). Moreover, a higher dose of norepinephrine was used in patients with abnormal RV-TI, with a median of 0.12 μg/kg/min (p = 0.02). This trend continued at 24 hours, with a median of 0.19 μg/kg/min (p = 0.02). Similarly, at 6 hours, there was a higher requirement for vasopressin in the abnormal RV-TI group, initiated in 41.3% of cases compared with 24.2% in the normal RV-TI group (p = 0.02). Regarding the requirement for inotropic support, at 24 hours it was observed that the group of patients with abnormal RV-TI required a higher dose of dobutamine, with a median of 6 μg/kg/min (p = 0.04). At 6 hours, there was also a higher frequency of methylene blue (13%) and steroid (10.9%) requirements in patients with abnormal RV-TI (p = 0.01 and 0.03, respectively) (**Table 3**).

**Table 3:** Hemodynamic parameters and vasoactive drugs

Variable	6 hours				24 hours			
	Total (n = 195)	RV-TI <0.53 (n = 149)	RV-TI ≥0.53 (n = 46)	p value	Total (n = 195)	RV-TI <0.53 (n = 149)	RV-TI ≥0.53 (n = 46)	p value
Cardiac index (L/min/m^2^), median (IQR)	2.1 (1.6–2.6)	2.1 (1.6–2.6)	2.2 (1.6–2.6)	0.86	2.2 (1.9–2.6)	2.2 (1.9–2.6)	2.2 (1.9–2.7)	0.84
Central venous pressure (mmHg), median (IQR)	9 (8–11)	9 (8–11)	10 (8–12)	0.24	11 (8–12)	11 (8–12)	11 (7–14)	0.97
Systemic vascular resistance index (dynes/s/cm^5^/m^2^), median (IQR)	2479 (1946–3159)	2544 (1954–3212)	2364 (1946–3007)	0.40	2394 (1942–2973)	2428 (1974–2973)	2229 (1810–2865)	0.29
Mixed venous O_2_ saturation (%), median (IQR)	67 (60–74)	67 (61–74)	65 (58–73)	0.34	67 (61–73)	68 (62–74)	64 (60–69)	<0.001
O_2_ extraction ratio (%), median (IQR)	32 (25–39)	32 (25–38)	32 (25–45)	0.23	31 (25–37)	30 (24–36)	33 (30–38)	<0.001
Venous-to-arterial CO_2_ pressure difference (mmHg), median (IQR)	7 (5–9)	7 (5–9)	7 (5–10)	0.72	6 (4–8)	6 (4–8)	7 (6–8)	0.20
Venous-to-arterial CO_2_ to arterial-to-venous O_2_ content difference ratio, median (IQR)	1.5 (1.1–2.1)	1.5 (1.2–2.1)	1.5 (0.9–2)	0.28	1.4 (1.1–2)	1.4 (1.1–2)	1.5 (1.1–2)	0.97
Lactate, median (IQR)	2.5 (1.7–4.1)	2.5 (1.9–4)	2.2 (1.5–5.4)	0.35	2.1 (1.5–2.8)	2 (1.5–2.6)	2.5 (1.7–3.7)	0.01
Norepinephrine, N (%)	110 (56.4)	77 (51.7)	33 (71.7)	0.01	40 (20.6)	27 (18.1)	13 (28.9)	0.11
Dose of norepinephrine, median (IQR)	0.08 (0.05–0.15)	0.08 (0.05–0.14)	0.12 (0.06–0.2)	0.02	0.11 (0.02–0.2)	0.05 (0.02–0.18)	0.19 (0.1–0.35)	0.02
Dobutamine, N (%)	62 (31.8)	43 (28.9)	19 (41.3)	0.11	24 (12.4)	16 (10.7)	8 (17.8)	0.20
Dose of dobutamine, median (IQR)	4 (3–6)	3.7 (2.5–6.1)	5 (3.6–6.2)	0.07	4 (3–5)	3 (3–5)	6 (3.5–12.5)	0.04
Vasopressin, N (%)	55 (28.2)	36 (24.2)	1 (41.3)	0.02	31 (16)	20 (13.4)	11 (24.4)	0.07
Dose of vasopressin, median (IQR)	0.05 (0.04–0.07)	0.05 (0.04–0.07)	0.05 (0.03–0.08)	0.75	0.06 (0.03–0.08)	0.06 (0.02–0.06)	0.08 (0.04–0.1)	0.18
Levosimendan, N (%)	36 (18.5)	27 (18.1)	9 (19.6)	0.82	26 (13.4)	18 (12.1)	8 (17.8)	0.32
Dose of levosimendan, median (IQR)	0.1 (0.1–0.1)	0.1 (0.1–0.1)	0.1 (0.1–0.1)	0.66	0.1 (0.1–0.1)	0.1 (0.1–0.1)	0.1 (0.08–0.1)	0.65
Milrinone, N(%)	2 (1)	1 (0.7)	1 (2.1)	0.41	4(2.1)	3 (2)	1 (2.2)	1
Dose of milrinone, median (IQR)	0.8 (0.7–1)	0.7 (0.7–0.7)	1 (1–1)	0.31	0.5 (0.1–0.7)	0.3 (0.07–0.6)	1 (1–1)	0.15
Methylene blue, N (%)	10 (5.1)	4 (2.7)	6 (13)	0.01	5 (2.6)	4 (2.7)	1 (2.2)	1
Steroids, n (%)	9 (4.6)	4 (2.7)	5 (10.9)	0.03	7 (3.6)	4 (2.7)	3 (6.7)	0.20

RV-TI: right ventricular Tei index; IQR: interquartile range

### Outcomes

The Sequential Organ Failure Assessment (SOFA) score was calculated at 24 and 72 hours, showing a significant difference between groups, particularly at 72 hours, with patients with abnormal RV-TI presenting a higher median score compared with those with normal RV-TI (p <0.001). Patients with abnormal RV-TI had a higher frequency of delirium (22.2% versus 8.7%, p = 0.01), in-hospital pneumonia (22.2% versus 7.4%, p ≤0.001), transfusion (66.7% versus 40.9%, p ≤0.001), and renal replacement therapy (15.6% versus 3.4%, p ≤0.001). A higher percentage of in-hospital mortality was also recorded among patients with abnormal RV-TI (17.8% versus 3.4%, p ≤0.001) (**Table 4**).

**Table 4:** Outcomes

Variable	Total (n = 195)	RV-TI <0.53 (n = 149)	RV-TI ≥0.53 (n = 46)	p value
Days in intensive care unit, median (IQR)	3 (2–4)	3 (2–4)	3 (2–5)	0.94
Days with mechanical ventilation, median (IQR)	1 (1–1)	1 (1–1)	1 (1–2)	0.02
SOFA score at 24 hours, median (IQR)	4 (3–6)	4 (3–6)	4 (3–7)	0.09
SOFA score at 72 hours, median (IQR)	3 (2–4)	2 (2–4)	4 (2–5)	<0.001
Delirium, N (%)	23 (11.9)	13 (8.7)	10 (22.2)	0.01
Stroke, N (%)	4 (2.1)	4 (2.7)	0	0.57
In-hospital pneumonia, N (%)	21 (10.8)	11 (7.4)	10 (22.2)	<0.001
Mediastinitis, N (%)	8 (4.1)	4 (2.7)	4 (8.9)	0.08
Transfusion, N (%)	91 (46.9)	61 (40.9)	30 (66.7)	<0.001
Acute kidney injury, N (%)	74 (38.1)	57 (38.3)	17 (37.8)	0.95
Renal replacement therapy, N (%)	12 (6.2)	5 (3.4)	7 (15.6)	<0.001
Liver injury, N (%)	45 (23.2)	36 (24.2)	9 (20)	0.56
Postsurgical atrial fibrillation, N (%)	31 (16)	24 (16.1)	7 (15.6)	0.92
Mortality, N (%)	13 (6.7)	5 (3.4)	8 (17.8)	<0.001

RV-TI: right ventricular Tei index; IQR: interquartile range

In the univariate analysis, an abnormal RV-TI (≥0.53) was significantly associated with higher risks of in-hospital mortality (OR 6.22, 95% CI 1.92–20.14, p = 0.002), pneumonia (OR 3.58, 95% CI 1.40–9.11, p = 0.007), delirium (OR 2.98, 95% CI 1.21–7.38, p = 0.018), renal replacement therapy (OR 5.30, 95% CI 1.59–17.64, p = 0.007), and transfusion (OR 2.88, 95% CI 1.43–5.81, p = 0.003). After adjustment for age, sex, and diabetes in the multivariate logistic regression model, these associations remained statistically significant, confirming that an RV-TI ≥0.53 independently predicts adverse outcomes (**Table 5**).

**Table 5:** Univariate and multivariate logistic regression analysis of adverse outcomes associated with RV-TI ≥0.53

Variable	OR	95% CI	p value
Univariate			
Mortality	6.22	1.92–20.14	0.002
In-hospital pneumonia	3.58	1.40–9.11	0.007
Delirium	2.98	1.21–7.38	0.018
Renal replacement therapy	5.3	1.59–17.64	0.007
Transfusion	2.88	1.43–5.81	0.003
Multivariate			
Mortality	5.48	1.62–18.58	0.006
In-hospital pneumonia	3.21	1.21–8.53	0.019
Delirium	2.74	1.08–6.95	0.034
Renal replacement therapy	4.87	1.42–16.73	0.012
Transfusion	2.55	1.24–5.25	0.011

Multivariate models were adjusted for age, sex, and diabetes.

OR: odds ratio; CI: confidence interval; RV-TI: right ventricular Tei index

## Discussion

This study provides valuable insights into RV-TI as a predictor of adverse clinical outcomes among cardiovascular surgery patients. We observed a higher prevalence of abnormal RV-TI among patients with diabetes mellitus and older age, suggesting its potential utility as a marker for identifying individuals at increased risk of postoperative complications and poorer clinical outcomes. Patients with abnormal RV-TI also demonstrated a higher incidence of immediate postoperative complications, including vasoplegic syndrome. They exhibited a less favorable hemodynamic profile characterized by lower mixed venous O_2_ saturation and elevated serum lactate levels.

Despite similar surgical durations between groups, patients with abnormal RV-TI required greater quantities and higher doses of inotropic and vasopressor support shortly after surgery. This increased need for hemodynamic support underscores the presence of underlying myocardial dysfunction and hemodynamic instability associated with abnormal RV-TI. In terms of clinical outcomes, we observed that these patients had higher SOFA scores at 72 hours, along with increased incidences of delirium, nosocomial pneumonia, need for transfusion, renal replacement therapy, and a significantly higher in-hospital mortality rate compared with those with normal RV-TI.

In our cohort, overall LV systolic function was preserved, with median LVEF values of 56% (IQR 48–60) across groups, and the majority of patients exhibited only mild to moderate diastolic dysfunction. Conventional echocardiographic parameters of RV function, including TAPSE (median 21 mm, IQR 18–23), FAC (median 43%, IQR 36–50), and S wave (median 11 cm/s, IQR 9.6–12.5), did not differ significantly between outcome groups. These findings suggest that conventional RV metrics may be relatively insensitive in detecting subtle or early RV dysfunction that contributes to adverse outcomes in this population. The RV-TI, by integrating both systolic and diastolic performance, provides a more comprehensive assessment of global RV function, which may explain its superior discriminative power. Given that LV systolic function was largely preserved, it is plausible that subclinical or overt RV dysfunction was a key driver of outcomes in our cohort, highlighting the clinical relevance of RV-TI as a prognostic marker.

Despite the absence of changes in macrohemodynamic parameters (cardiac index and CVP), our study found marked alterations in global oxygenation indices (mixed venous O_2_ saturation and O_2_ extraction ratio), as well as in the perfusion marker (serum lactate levels). This suggests that microcirculatory disturbances may be present in these patients, rather than changes in macrohemodynamics, which could be compensated by the higher use and doses of vasoactive drugs observed in our cohort. This is clinically relevant because, although macrohemodynamic targets are maintained, tissue oxygenation remains impaired, indicating persistent abnormalities at the capillary–cell interface.^12)^

While these findings highlight RV-TI as a sensitive marker of RV dysfunction and adverse outcomes, it is not intended to replace established risk scores such as STS or EuroSCORE II. Instead, RV-TI serves as a complementary tool that provides detailed information during preoperative risk assessment, helping to identify high-risk patients who may benefit from targeted perioperative management. Its integration into clinical practice can enhance the detection of subtle RV dysfunction and help guide individualized strategies to optimize hemodynamic support, ultimately improving postoperative outcomes and reducing the associated morbidity and mortality.

### Limitations

Despite the noteworthy findings obtained, it is crucial to acknowledge several potential limitations inherent to this study:

• The sample size was relatively small, which may limit the generalizability of the findings to a broader population. A larger cohort could provide a more comprehensive understanding of the relationship between RV-TI, vasoplegic syndrome, and in-hospital mortality.
• The retrospective design introduces the possibility of selection and information biases that could influence the observed outcomes. A prospective study design would offer a more precise assessment of the causal relationships between the variables studied.
• Measurement of the RV-TI could be affected by inter-observer variability and variations in measurement technique, potentially introducing errors in the estimation of cardiac function.
• The incidence of AKI did not differ between cohorts. This is likely explained by our registry definition, since AKI was classified from KDIGO stage 1 (serum creatinine increase ≥0.3 mg/dL within 48 hours, or ≥1.5 times baseline within 7 days, or urine output <0.5 mL/kg/h for 6 hours). We assume that patients with impaired RV-TI in our cohort experienced higher rates of more severe AKI. In our center, indications for renal replacement therapy include persistent oliguria despite high-dose loop diuretics (240 mg furosemide/24 hurs or 6 mg bumetanide, bolus or infusion), blood urea nitrogen >100 mg/dL, refractory metabolic acidosis (pH <7.2), or refractory volume overload.
• The study primarily focused on in-hospital outcomes and did not include long-term follow-up of patients after discharge. This limits insights into the enduring impacts of the RV-TI on morbidity and mortality beyond the immediate postoperative period.

Despite these limitations, the findings of this study provide a solid foundation for future investigations and underscore the importance of considering RV-TI as a potential predictor of complications and outcomes in patients undergoing cardiac surgery.

## Conclusion

Integrating RV-TI assessment into clinical practice as a tool for risk stratification can yield significant benefits by facilitating early identification of high-risk patients, preventing serious complications, and ultimately improving clinical outcomes and postoperative survival among cardiac surgery patients.

## Declarations

### Data availability

The data supporting the findings of this study are available upon reasonable request from the corresponding author (DM-S).
